# Impact of better and worse eye damage on quality of life in advanced glaucoma

**DOI:** 10.1038/srep04144

**Published:** 2014-02-20

**Authors:** Mizu Okamoto, Kenji Sugisaki, Hiroshi Murata, Hiroyo Hirasawa, Chihiro Mayama, Ryo Asaoka

**Affiliations:** 1 Department of Ophthalmology, the University of Tokyo Graduate School of Medicine, Tokyo, Japan; 2 Tokyo Koseinenkin Hospital, Tokyo, Japan

## Abstract

The purpose of the study was to investigate the influence of VF and the VA on vision related quality of life (VRQoL) in advanced glaucoma. Subjects consist of 50 glaucoma patients with mean deviation (MD) less than −20 dB in at least one eye. Patients' VRQoL was assessed using the ‘Sumi questionnaire’. The impact of seven visual measures on VRQoL were compared using principal component regression: MDs of better and worse eyes with 10-2 and 24-2 Humphrey VFs, LogMAR VAs of better and worse eyes and the Esterman score. The root mean of the squared prediction error (RMSE) was calculated using leave-one-out cross validation. Better eye summary measurements were much more influential on VRQoL than corresponding worse eye measurements and Esterman score in every VRQoL task. In conclusion, in advanced glaucoma, VF parameters of the better eye are important for the VRQoL of the patient.

Glaucoma is one of the leading causes of blindness worldwide^1^,^2^. In glaucoma, visual field (VF) loss^3^,^4^,^5^,^6^,^7^,^8^,^9^,^10^,^11^,^12^ and reduced visual acuity (VA)^9^,^10^,^11^,^12^,^13^,^14^,^15^ impact on patients' vision-related quality of life (VRQoL), which can be defined as a patient's satisfaction with their visual ability and how their vision impacts on their daily life^16^. Furthermore, glaucomatous VF deterioration can impair hand-eye coordination^17^, can increase the risk of falling^18^, and can increase the likelihood of causing or being involved in a motor vehicle accident^18^,^19^,^20^,^21^,^22^ likely because of the inability to detect peripheral obstacles and hazards^20^,^23^.

A number of studies have examined the relationship between VRQoL and patients' VF impairments^3^,^4^,^8^,^24^,^25^. However, many of the patients investigated in these studies had early stage disease. In addition, we previously reported that different areas of the VF are important for various daily tasks in a population of glaucoma patients with a wide range of VF damage^26^. Glaucomatous VF change usually starts in the midperipheral VF, while the central region tends to be spared until late on in the disease process^27^. Consequently, in advanced glaucoma, patients' functional VFs are often constricted to the central island near fixation^2^. However, the stage of glaucomatous disease can vary considerably between eyes in a patient, which makes clinical treatment decisions difficult. Thus, our motivation in this study was to investigate which eye in patients with advanced glaucoma has greatest impact on VRQoL. This is in contrast with our previous study that investigated which areas of the integrated (binocular) visual field are most important for quality of life^26^.

There is no study which elucidated which eye of a patient (worse eye or better eye) has a larger impact on VRQoL in advanced glaucoma. Moreover, previous research has been based on only one VF test pattern, such as the Esterman test, 30-2 test, or 24-2 test of the Humphrey Field Analyzer (Zeiss-Humphrey Systems, Dublin, CA [HFA])^3^,^4^,^8^,^24^. To date, no research has investigated both central and peripheral VF results simultaneously in patients with advanced glaucoma (defined here as a 24-2 mean deviation (MD) less than −20 dB in the worse eye). In the current study, 10-2 and 24-2 VFs were carried out alongside the Esterman binocular VF test and VA measurements to investigate which eye has the larger impact on VRQoL in patients with advanced glaucoma.

## Results

**Figure 1:**
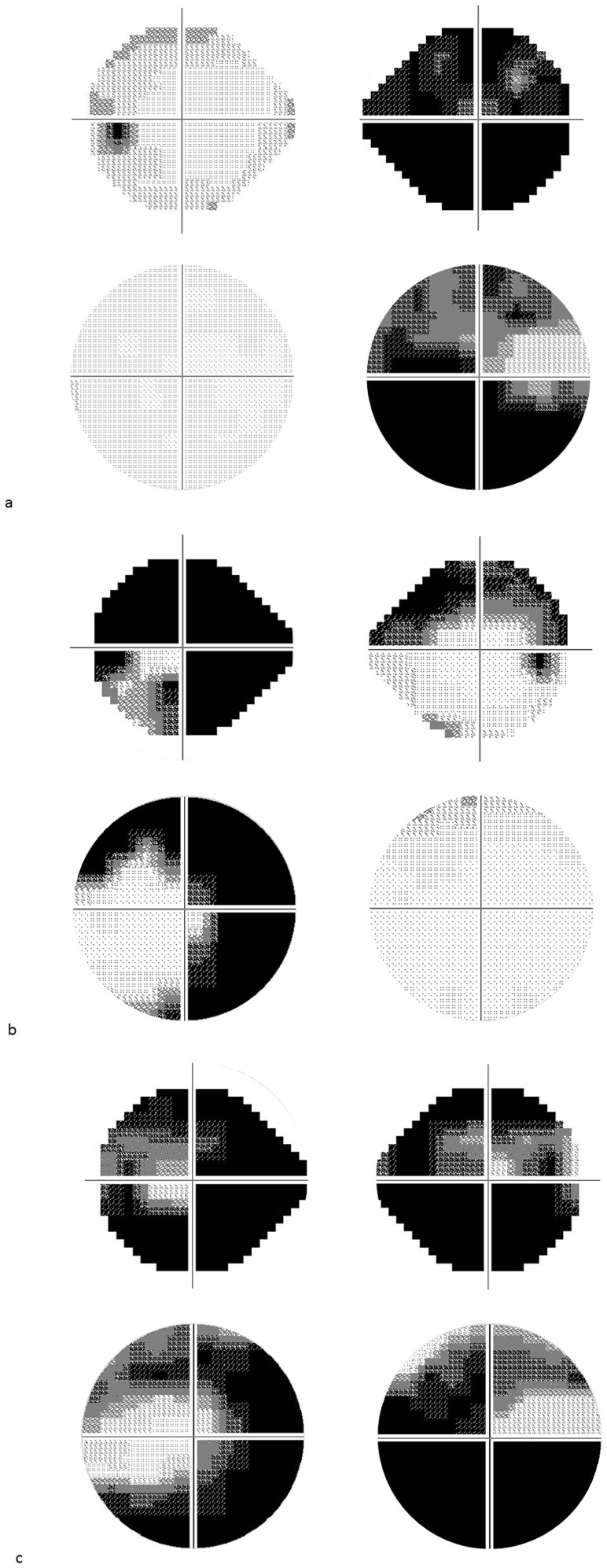
24-2 VF and 10-2 VF of sample cases. Figure 1(a), Grayscales of case a (80 year old, male). MDs of the right eye were −29.6 (24-2 VF) dB and −26.5(10-2 VF) dB and those of the left eye were −4.0 (24-2 VF) and −2.0 dB (10-2 VF). Figure 1(b), Grayscales of case b (72 year old, male). MDs of the right eye were −10.0 (24-2 VF) dB and −0.7(10-2 VF) dB and those of the left eye were −27.7 (24-2 VF) and −21.5 dB (10-2 VF). Figure 1(c), Grayscales of case c, (78 year old, female). MDs of the right eye were −27.2 (24-2 VF) dB and −27.8(10-2 VF) dB and those of the left eye were −27.6 (24-2 VF) and −23.9 dB (10-2 VF). MD: mean deviation, VF: visual field.

**Figure 2:**
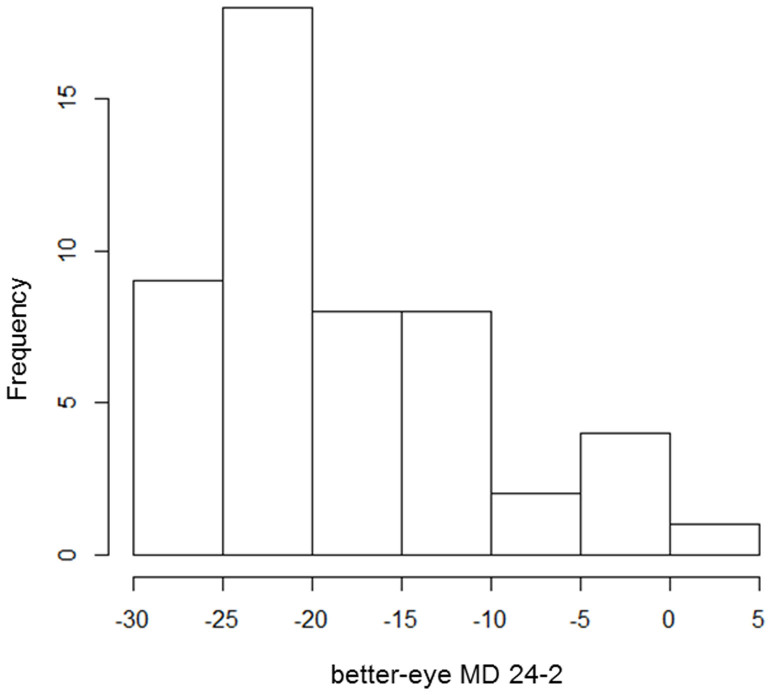
Histogram of better-eye MD 24-2.

Subject demographics are given in Table 2. Figure 1 shows the grayscale plots of the 24-2 VF and 10-2 VF in three example patients. The distribution of the better-eye MD 24-2 is shown in Figure 2.

The relationships between each VRQoL score and better-eye VA, worse-eye VA, better-eye MD 24-2, worse-eye MD 24-2, better-eye MD 10-2 and worse-eye MD 10-2 are shown in the 3D scatter plots (Figure S1, S2, S3, S4 and S5).

**Figure 3:**
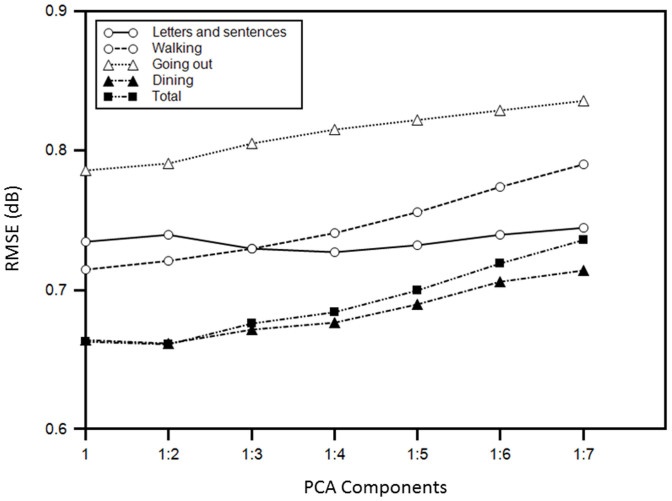
RMSE with first to seventh PCA components. RMSE was obtained using the leave-one-out cross validation method and standard linear regression. Adding additional PCA components to the first component did not significantly decrease RMSE (Wilcoxon test, p ≥ 0.05). RMSE: root mean of the squared prediction error, PCA: principal component analysis.

Figure 3 shows RMSEs for each VRQoL score; RMSE generally increased with an increase in the number of PCA components; however, there was not a significant decrease in RMSE when additional components were added to the first PCA component (Wilcoxon test, p ≥ 0.05).

Table 3 shows the loading values of the first PCA component for each VRQoL score. Absolute loading values were larger for better eye measurements compared with the corresponding worse eye measurements in every VRQoL task. The loading values of 10-2 VF MDs and 24-2 VF MDs were very similar in each eye, and these tended to be larger than VA absolute loading values. Furthermore, for each task, the absolute loading value for the Esterman score was smaller than the absolute loading values for better-eye MD 24-2 and better-eye MD 10-2, and only slightly larger than the better-eye VA value. Loading values for age were much smaller than all other parameters.

## Discussion

In the current study, VAs, 24-2 VFs, 10-2 VFs and Esterman VFs were measured in patients with advanced stage glaucoma, along with VRQoL questionnaires. Our group has previously reported that different VF areas are important for different daily tasks; however, our previous study was carried out in a sample of patients with a range of VF damage from early to advanced stage glaucoma. The motivation of the current research was to investigate which eye (better or worse eye) in a patient has the largest impact on VRQoL in glaucoma; we restricted our study population to patients with advanced glaucoma in at least one eye because the stage of VF damage can considerably vary between eyes in a patient, which makes clinical treatment decisions difficult. PCR was carried out in order to investigate the importance of different vision measurements on VRQoL. It was observed that the first PCA component adequately minimized RMSE for all VRQoL tasks. Thus, the loading values of the first PCA components were calculated and it was found that vision measurements (VA, 24-2 VF MD and 10-2 VF MD) of the better eye had larger impact on all of VRQoL scores, including total score.

A previous report concluded that visual function in the central VF is predominantly important for VRQoL^29^; in the study, the impact of various VF sensitivity measurements were compared using Pearson's correlation test and multiple regression analysis. As already pointed out, these methods can be affected by the problem of multicollinearity. We have recently suggested that VRQoL can be better predicted when point-wise VF sensitivities and VA are investigated simultaneously, using the Random Forest machine learning method. Moreover, we showed that peripheral VF areas were no less important for various VRQoL tasks than the central area^26^. Indeed, other recent studies have also postulated the importance of the peripheral VF for VRQoL tasks, such as driving^23^ and maintaining postural stability^37^. The results of our current study suggest that MD of the 24-2 VF is no less important than MD of the 10-2 VF for all VRQoL tasks.

Many previous studies^8^,^10^,^12^,^29^,^38^ have tried to ascertain whether VA of the better eye or VA of the worse eye is more important for VRQoL. One of the problems with these previous reports is that the influence of VA and VF measurements were investigated independently for better and worse eyes. One study did analyze VAs of better and worse eyes simultaneously using a multiple linear regression model and concluded that VA of the worse eye is more important for VRQoL^4^. However, multiple linear regression cannot overcome the problem of multicollinearity and hence, more appropriate statistical methods should be applied before drawing any such conclusion. Furthermore, importantly, these results contradict the conclusions of our previous report in that VA of the worse has a stronger impact on all VRQoL scores than the VA of better eye^26^. These inconsistent results may be attributed to the problem of multicollinearity, or, may be a result of the different populations of subjects studied; the mean ± standard deviation of MD in the 24-2 VF was −1.5 ± 2.5/−4.9 ± 3.7 dB in the better/worse eye^4^ in the Sumi et al. study^29^, and −13.1 ± 9.3/−17.9 ± 9.6 dB in the better/worse eye^26^ in our previous research. In general, VRQoL deterioration is closely related to VA of the worse eye; however, in patients with advanced glaucoma, the VF of the better eye may be more closely related to VRQoL.

Many previous reports have used the integrated (binocular) VF (IVF) to investigate the relationship between VRQoL and a patient's VF^23^,^39^,^40^,^41^. Indeed, it has been reported that the discrepancy between a glaucoma patient's better eye MD and worse eye MD becomes more pronounced with progression of the disease^16^. In the current study, the agreement between the binocular and monocular MDs was very high; binocular-better MD 24-2 and better-eye MD 24-2(R = 0.96, p < 0.01). This is because, in the current study, most binocular total deviation values were taken from a patient's better eye, because the worse eyes were very advanced and so most VF sensitivities approached the bottom of the range. Actually the definition of advanced glaucoma in the current study is more strict than the classification by Anderson et al^42^. As a result, most total deviation values of the IVF were taken from the better-eye, which results in only marginal differences between the MDs of IVFs and MDs of better eyes. Thus, the argument of whether to use the IVF or monocular VFs to predict VRQoL is less applicable to the current study.

A previous report by Jampel and colleagues suggested monocular VFs are more relevant to patients' VRQoL than the Esterman VF^25^. Our results are in agreement with this finding; as indicated in Table 3, the Esterman score's absolute loading value larger was smaller than both monocular indices for all VRQoL scores, despite the larger number of test points (n = 120) in the Esterman test compared to the 24-2 VF (n = 52). Moreover, the Esterman test has much wider coverage (more than 130 degrees) than the 24-2 VF and has more test points concentrated in the central area, which are reported to be beneficial when predicting VRQoL^29^,^43^. In addition, in contrary to the integrated VF, the Esterman test is an actual measure of the binocular VF, rather than an estimate of it, and therefore it has been used in many studies to evaluate the relationship between patients' VFs and VRQoL^6^,^11^,^44^,^45^,^46^,^47^,^48^. Also, the Esterman test fails to measures the entire central six degrees. We propose that the Esterman score was less important to predict VRQoL on the current study because it is merely a suprathreshold test. Thus, our findings suggest that it may not be necessary to carry out an extra-measurement of the Esterman VF, in this population of advanced glaucoma patients, to predict VRQoL.

A possible caveat of the current study is that localized VF damage is inadequately described by the MD measurement. Our group has already suggested that VRQoL can be predicted more accurately when point-wise VF sensitivity is used compared to the global index of MD^26^. The relationship of point-wise VF sensitivities in the 10-2 and 24-2 VFs, along with VA should be carried out in a future study.

In conclusion, we have shown that MDs of the 10-2 and 24-2 VFs and VA in patients' better eyes are more influential than the corresponding measurements in patients' worse eyes on VRQoL, in patients with advanced glaucoma. VF parameters are not only important for assessing the severity of glaucoma, but also the VR-QoL of the patient. Indeed, our results suggest that, for assessing VR-QoL in patients with advanced glaucoma, VF parameters are more relevant than VA measurements. This finding is important to help clinicians make more timely interventions and maintain VR-QoL in advanced glaucoma patients.

## Methods

The study was approved by the Research Ethics Committee of the Graduate School of Medicine and Faculty of Medicine at the University of Tokyo. Informed consent was obtained from all subjects. This study was performed according to the tenets of the Declaration of Helsinki.

The better and worse eyes were decided by comparing MD values from the 24-2 VF test. This study included 50 patients from the glaucoma clinic at the University of Tokyo Hospital (mean ± standard deviation (sd) age was 61 ± 13 years, [range: 36–83]) with a definitive diagnosis of primary open-angle glaucoma (25 patients), normal tension glaucoma (16 patients), primary angle-closure glaucoma (2 patients), secondary open angle glaucoma (7 patients). All patients enrolled in the study fulfilled the following criteria: (1) glaucoma was the only disease causing VF damage and/or VA impairment; (2) patients were followed for at least 6 months at the University of Tokyo Hospital; (3) patients' intraocular pressure was less than 21 mmHg at the time of VF measurements and VRQoL interview; (4) at least one eye had glaucomatous VF damage in the advanced stage; MD < −20 dB in the 24-2 VF.

All of the VF measurements and VRQoL interview were carried out within a period of six months. 24-2 and 10-2 VFs were measured with the HFA (Swedish Interactive Threshold Algorithm standard program) and a Goldmann size III target. Only VFs with fixation losses < 25% and false-positive error < 15% were analyzed; false negative rate was not used to exclude VFs.^28^. In addition, the Esterman test was carried out using the HFA. Corrected VA was measured using the Landolt C chart and LogMAR was used for subsequent analyses. Following the methodology employed in our previous paper^26^, VRQoL was assessed using the method originally developed by Sumi et. al^26^,^29^. The ‘Sumi Questionnaire’ is written in Japanese and contains 30 questions regarding 7 tasks: legibility of letters (‘letters’), legibility of sentences (‘sentences’), walking, using public transportation (‘going out’), dining, dressing, and miscellaneous activities (‘miscellaneous’) (Table 1). Questions were translated into English for this paper). The Sumi questionnaire includes one question (question 7) about reading vertically, because this is the traditional way to read and write in Japanese. Each response was scored as follows; greatly disabled (2 points), slightly disabled (1 point), and not disabled (0 points). Mean score was calculated for each of the seven tasks to attain a visual disability index. The ‘letters’ and ‘sentences’ scoreswere combined because these tasks are closely correlated. The ‘dressing’ task was not analyzed because of the small number of questions (n = 2), but its score was used in the calculation of overall VRQoL.

The relationship between each VRQoL score and the VAs of better and worse eyes (better-eye VA and worse-eye VA), 24-2 VF MDs of better and worse eyes (better-eye MD 24-2 and worse-eye MD 24-2), 10-2 VF MDs of better and worse eyes (better-eye MD 10-2 and worse-eye MD 10-2) and the Esterman score was investigated using principal component regression (PCR). In this technique, principal component analysis (PCA) is carried out between each of the six VRQoL scores (each of the five tasks and the total VRQoL score), and better-eye VA, worse-eye VA, better-eye MD 24-2, worse-eye MD 24-2, worse-eye MD 10-2, worse-eye MD 10-2 and the Esterman score. Then using the leave-one-out cross validation method^30^, the root mean of the squared prediction error (RMSE) was calculated. In this cross validation, 50 patients are divided into learning (49 patients) and testing (one patient) datasets and the VRQoL score of the test patient is predicted using the PCA components obtained from the learning dataset, using standard linear regression. Next, the prediction error was calculated as (predicted VRQoL score) – (actual VRQoL score). This process was repeated 50 times so that all of the 50 patients were used as test data once. RMSEs were calculated using the first to seventh PCA components. The optimum number of components was then decided based on the point at which RMSE ceased to significantly decrease; finally, the loading values of the selected PCA components were calculated.

Multicollinearity is a statistical phenomenon in which predictor variables in standard linear regression model are correlated. In this situation, standard linear regression can give biased results for individual predictors, because the variance of some of the estimated regression coefficients can become very large. In the current study, MDs of the 10-2 VF and 24-2 VF are related^31^, as one would expect due to the fact that some 10-2 VF test points are a subset of the 24-2 VF^32^,^33^. Furthermore, the sensitivities of VF test points in the central VF are strongly correlated with VA^34^, because of their location close to fixation. Finally, we frequently find that MDs of better and worse eyes in a single patient are correlated. Thus, it is not appropriate to carry out standard linear regression in this research. The problem of multicollinearity, however, is not applicable to PCR, because PCA components are independent to each other^35^, PCR is therefore commonly used to overcome this problem^36^.

All statistical analyses were carried out using the statistical programming language R (ver. 2.15.0, The R Foundation for Statistical Computing, Vienna, Austria).

## Supplementary Material

Supplementary Information Figure S

## Figures and Tables

**Table 1:** Questions included in the ‘Sumi Questionnaire’ (questions originally written in Japanese)

**Legibility of letters: letters**
1. Can you read the headlines of a newspaper? (Yes/With difficulty/No)
2. Can you read small print in a newspaper? (Yes/With difficulty/No)
3. Can you read words in a dictionary? (Yes/With difficulty/No)
4. Can you see the numbers in a telephone directory? (Yes/With difficulty/No)
5. Can you make out a fare table for trains and subways? (Yes/With difficulty/No)
**Sentences**
6. Do you have difficulty reading and writing? (No/Occasionally/Frequently)
7. When you write sentences in vertical lines, does it lean to either
direction?
(No/Occasionally/Frequently)
8. When you read, can you find the next line easily? (Yes/With difficulty/No)
**Walking**
9. Do you have difficulty walking because of your visual problems? (No/Occasionally/Frequently)
10. Can you take a walk by yourself? (Yes/With difficulty/No)
11. Do you misjudge traffic signals? (No/Occassionally/Frequently)
12. Do you bump into people or objects while walking? (No/Occasionally/Frequently)
13. Do you stumble on the stairs? (No/Occasionally/Frequently)
14. Do you fail to notice changes in the ground? (No/Occasionally/Frequently)
15. Do you fail to recognize your friends until they talk to you? (No/Occasionally/Frequently)16. Do you fail to see people or cars approaching you from the side? (No/Occasionally/Frequently)
**Going out**
17. Do you have difficulty going out because of your visual problems? (No/Occasionally/Frequently)
18. Do you need somebody to accompany you to go to new places? (No/Preferably/Yes)
19. Can you get a cab by yourself? (Yes/With difficulty/No)
20. Do you have difficulty traveling by train? (No/Occasionally/Frequently)
21. Do you feel uneasy going out at night because of your visual problems? (No/Occasionally/Frequently)
**Dining**
22. Do you have difficulty dining because of your visual problems? (No/Occasionally/Frequently)
23. Do you drop food while dining because of your visual problems? (No/Occasionally/Frequently)
24. Do you spill tea while pouring into a cup? (No/Occasionally/Frequently)
25. Do you have difficulty using chopsticks? (No/Occasionally/Frequently)
**Dressing**
26. Do you ever button up clothing in the wrong order? (No/Occasionally/Frequently)
27. Can you see your face clearly in the mirror? (Yes/With difficulty/No)
**Miscellaneous**
28. Can you recognize people's faces on TV? (Yes/With difficulty/No)
29. Do you have difficulty finding objects dropped on the floor? (No/Occasionally/Frequently)
30. Do you have difficulty dialing the telephone? (No/Occasionally/Frequently)

**Table 2:** Subject demographics

Demographics	value
gender (male:female)	32:18
Age (years) (mean ± SD [range])	61 ± 13 [36 to 83]
better-eye MD 24-2 (dB)	−18.4 ± 7.6 [−29.0 to 0.4]
worse-eye MD 24-2 (dB)	−26.8 ± 2.5 [−32.1 to −20.9]
better-eye MD 10-2 (dB)	−16.7 ± 9.3 [−33.3 to −0.7]
worse-eye MD10-2 (dB)	−24.7 ± 4.9 [−34.4 to −13.7]
Esterman score (dB)	73.8 ± 17.1 [26 to 97]
better-eye VA (LogMAR)	0.05 ± 0.3 [−0.3 to 1.2]
Worse-eye VA (LogMAR)	0.1 ± 0.3 [−0.3 to 1.0]

**Table 3:** The loading values of PCA components in the PCR against various VRQoL scores

	letters and sentences	walking	going out	dining	Total
better-eye MD 24-2	−0.18	−0.14	−0.12	−0.18	−0.18
worse-eye MD 24-2	−0.08	−0.06	−0.06	−0.08	−0.08
better-eye MD 10-2	−0.17	−0.13	−0.12	−0.17	−0.17
worse-eye MD 10-2	−0.08	−0.06	−0.06	−0.08	−0.08
better-eye VA	0.11	0.08	0.08	0.11	0.11
worse-eye VA	0.05	0.04	0.03	0.05	0.05
Esterman score	−0.14	−0.10	−0.10	−0.14	−0.14
age	0.02	0.01	0.01	0.02	0.02

PCA: principal component analysis, PCR: principal component regression, MD: mean deviation, better-eye MD 24-2: MD of better 24-2 visual field, worse-eye MD 24-2: MD of worse 24-2 visual field, better-eye MD 10-2: MD of 10-2 visual field in the eye of better MD with the 24-2 visual field, worse-eye MD 10-2: MD of 10-2 visual field in the eye of worse MD with the 24-2 visual field, better-eye VA: visual acuity in the eye of better MD with the 24-2 visual field, worse-eye MD 10-2: visual acuity in the eye of worse MD with the 24-2 visual field.

## Notes

Supported in part by Grand 60645000 (H.H.), 25861618 (H.M.), 50570701 (C.M.) from the Ministry of Education, Culture, Sports, Science and Technology of Japan.
