# Supplementary figures and images for: Impact of better and worse eye damage on quality of life in advanced glaucoma
Source: Sci Rep. 2014 Feb 20;4:4144. doi: 10.1038/srep04144 (PMC5379256; doi:10.1038/srep04144)

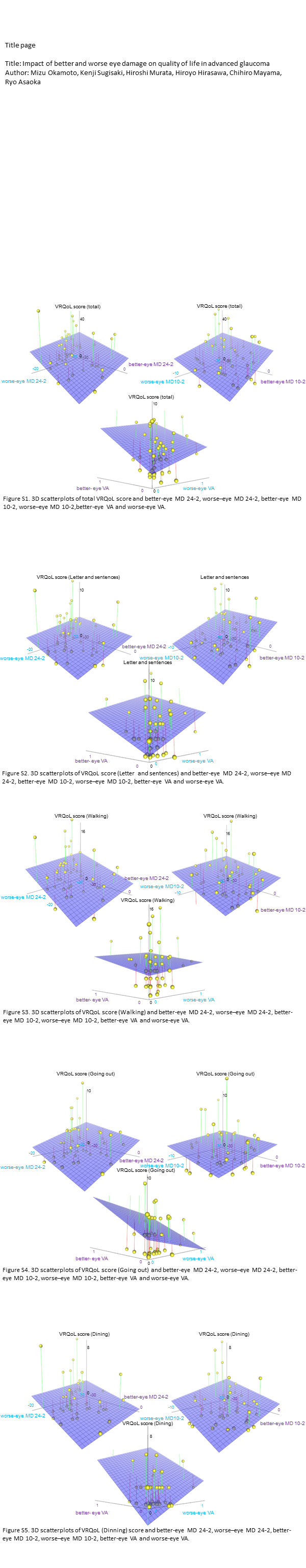

Supplement: Supplementary Information — Figure S [file srep04144-s1.tiff]
